# Regulatory T cell homing and activation is a signature of neonatal sepsis

**DOI:** 10.3389/fimmu.2024.1420554

**Published:** 2024-07-12

**Authors:** Darius Sossou, Sem Ezinmegnon, Gino Agbota, Komi Gbedande, Manfred Accrombessi, Achille Massougbodji, Marceline d’Almeida, Jules M. Alao, Ida Dossou-Dagba, Alexandre Pachot, Laurence Vachot, Karen Brengel-Pesce, Gilles Cottrell, Akadiri Yessoufou, Valérie Briand, Pierre Tissières, Nadine Fievet

**Affiliations:** ^1^ Paris-City University, Mére et Enfants en Milieu Tropical: pathogénes, systéme de santé et transition épidémiologique (MERIT), Institute of Research for Development (IRD), Paris, France; ^2^ Faculty of Sciences and Technology (FAST), University of Abomey-Calavi, Institute of Applied Biomedical Sciences (ISBA), Laboratory of Cell Biology and Physiology, Cotonou, Benin; ^3^ Institut de Recherche Clinique du Bénin (IRCB), Calavi, Benin; ^4^ Fédérations Hospitalo-Universitaires (FHU) Sepsis, AP-HP/Université Paris Saclay/Inserm, Le Kremlin-Bicêtre, France; ^5^ Pediatric Department, National University Hospital Center (CNHU), Cotonou, Benin; ^6^ Pediatric Department, Mother and Child University and Hospital Center (CHUMEL), Cotonou, Benin; ^7^ Pediatric Department, Calavi University Hospital, Calavi, Benin; ^8^ Medical Diagnostic Discovery Department, bioMérieux, Marcy l’Etoile, France; ^9^ Institute of Integrative Biology of the Cell (I2BC), Centre National de la Recherche Scientifique (CNRS), Commissariat à l'Energie Atomique et aux énergies alternatives (CEA), University Paris Saclay, Gif-sur-Yvette, France; ^10^ Pediatric Intensive Care and Neonatal Medicine, Assistance Publique - Hôpitaux de Paris (AP-HP) Paris Saclay University, Bicêtre Hospital, Le Kremlin-Bicêtre, France

**Keywords:** Treg, immunity, sepsis, newborn, prematurity, malaria in pregnancy

## Abstract

Regulatory T cells (Treg) play a prominent role *in utero* tolerating non-inherited maternal antigens and in regulating immune responses against pathogens at birth. This study investigates Treg immunity in newborns in West Africa, where sepsis remains a major public health problem. Treg phenotypes on neonates subgroups with early-onset sepsis (EOS), presumed sepsis, and healthy newborn with and without prenatal risk factors were evaluated. Treg phenotypes varied according to prenatal conditions, with increase in Treg frequency and Foxp3 expression in healthy newborns with prenatal risk factors compared to those with none risk. Compared to healthy newborns with prenatal risk factors, EOS neonates had a significantly reduced frequency of Treg and Foxp3 expression. In the Treg pool, higher frequency of activated Treg was observed in EOS neonates, suggesting an *in-utero* activation upstream of the sepsis onset. Their migration to the infection site may explain the reduced frequency of circulating Integrin α4β1^+^ Treg suggestive of homing to the endothelial tissue. EOS neonates show increases expression of CTLA-4, PD-1 and CD39 on Treg, which negatively regulate the activation of effector T cells (Teff) corroborating by the lower frequency of Teff in EOS neonates. The higher frequency of CD39^+^ Treg and the lower frequency of integrinα4β1^+^ Treg in EOS non-survivor suggests that Treg exhaustement and endothelial homing are associated with outcome severity. Neonates developing EOS are born with an altered Treg phenotypic profile. Treg expression of CTLA-4, PD-1, CD39, and integrinα4β1 cell markers can be considered as early warning or diagnostic markers of EOS.

## Introduction

Sepsis is a life-threatening organ dysfunction caused by a dysregulated host response to infection (1, 2). In 2020, an estimated 15% (approximately 3,9 million) of all neonatal deaths worldwide were due to sepsis (3). Sepsis stands as one of the primary causes of neonatal mortality, particularly affecting preterm and low-birth-weight infants. The highest burden of neonatal sepsis is in sub-Saharan Africa and South Asia, where nearly half of all neonatal deaths occur (4). Besides the elevated incidence of neonatal sepsis, sub-Saharan Africa and South Asia, which encompass 52% of world global live births, have an overweighed proportion of prematurity, which represents 60% of all preterm births worldwide (5, 6). Contextually, prematurity is an age-dependent known risk factor for developing neonatal sepsis. Parasitic infection during pregnancy, such as malaria in pregnancy, is also identified as affecting both the occurrence of premature delivery and neonatal immunity and is a risk factor for developing infections during the first year of life (7, 8).

Fetal immune maturation takes place during the last three months of pregnancy. The increased risk of neonatal infection and impaired host response observed in preterm infants is related to a functional immune system not adapted to an environment other than the placenta, resulting in innate and regulatory immune functions that do not allow adequate response to environmental pathogens (9, 10). Among other factors associated with neonatal infection, endemic parasitic infections such as malaria affect fetal and perinatal development, including the immune system. The sepsis pathophysiology is complex and highly dependent on immunological effectors mobilized to face an infection (1, 2). Immune regulatory mechanisms are central to an effective antimicrobial response. Those mechanisms may be initially beneficial to dampen overwhelming inflammation, but if persisting, it may lead to secondary immunodepression. The loss of effector cells and functions of immunoregulatory cellular subtypes characterizes sepsis-associated immunodepression. Treg’s anti-inflammatory response can lead to immunosuppression. This status impedes the elimination of the primary infection and promotes secondary nosocomial infections and the reactivation of dormant pathogens (2, 11).

Regulatory T cells play a prominent role *in utero* tolerating non-inherited maternal antigens and at birth, essential in the control of immune responses to pathogens (12–14). They are often defined by the surrogate markers CD4 and CD25, but the Forkhead box P3 (FOXP3) will extensively characterize the suppressive function of most Treg. The population and function of CD4^+^CD25^+^CD127^-^ Treg are considerably diverse, with phenotypically and functionally distinct subsets of Treg that mediate immune suppression through distinct mechanisms with different cell surface markers (15). The most well-known surface markers are the cytotoxic T lymphocyte-associated antigen-4 (CTLA-4), the programmed death-1 (PD-1) and its ligand (PD-L1), the ectonucleotidase CD39 and the glucocorticoid-induced TNFR-related protein (GITR) (16). CTLA-4 binds to CD80 and CD86 on antigen-presenting cells (APCs), especially dendritic cells (DCs), inhibits the antigen presentation and maturation function of APCs (17), and increases the expression of Indoleamine 2,3-dioxygenase in DCs, reducing the concentration of tryptophan necessary for effector T cell proliferation (18). PD-1 (CD279) binds to PD-L1 and PD-L2 ligands on DCs to inhibit effector T cells (19). Treg cells exert their functions through soluble intermediates. The extracellular and/or pericellular accumulation of adenosine causes an immunosuppressive response (20). CD39/CD73 expressed on Treg cells degrade ATP into adenosine, and the increase in the adenosine concentration in the microenvironment will inhibit antigen presentation by DCs (21). Two origins compartments have been described for FOXP3^+^ Treg: the thymus and the periphery. In the thymus, FOXP3^+^ T cells are generated by positive selection of conventional CD4^+^ T cells. In the periphery, several triggers induce the expression of FOXP3 in naïve CD4^+^ T cells (22). There is evidence that T cells with a naïve CD4^+^ CD25^+^ CD45RA^+^ surface profile and immunosuppressive properties are detectable within the peripheral Treg pool (23, 24). Unlike the antigen-primed Treg, this naïve subset may comprise *de novo*-generated cells recently released from the thymus and have not yet experienced antigen exposure. Surface expression of CD31 on naïve CD4^+^ T cells distinguishes recent thymic emigrants (RTEs) from peripherally expanded naïve T cells (CD31^-^) (25). RTEs, unlike the naïve T cells lacking CD31, contain high T cell receptor (TCR) excision circles (TRECs). TRECs are generated as a by-product of the TCR rearrangement process in the thymus and are enriched in newly generated T cells (26).

It has been documented that PD-1 and CTLA-4 might be associated with the severity and control of sepsis by upregulating Treg cells (27, 28) and that increasing CD39^+^Treg was associated with a poor prognosis for sepsis in adult patients (29). In addition, the percentage of CD31^-^Treg was significantly higher in cord blood of the preterms related to inflammation and prenatal lipopolysaccharide exposure. Thus, in recent years, one of the therapeutic strategies being considered for some patients is immunostimulation, which involves targeting immune alterations to restore an appropriate anti-infectious response (30, 31). The most documented was immuno-adjuvant therapy with anti-PD-1, anti-CTLA-4, and anti-Tim-3 antibodies, which can reverse sepsis-induced immunosuppression and improve survival in sepsis (27, 32).

Identifying neonates developing immunosuppression at birth is a prerequisite for the use and success of immunostimulation therapy. However, neonatal sepsis is a very heterogeneous disease regarding the nature of the immune status and the intensity and timing of the onset of immunoinflammatory disorders. In this context, the exhaustive description of immune alterations in neonates affected by prematurity and malaria in pregnancy (MiP) at birth is fundamental for better understanding neonatal sepsis’s pathogenesis. This could lead to identifying biomarkers for early sepsis diagnosis and therapeutic targets for immunotherapies. The immunological effectors associated with neonatal sepsis and other perinatal infectious factors, such as prematurity, have not been studied in malaria-endemic areas. According to the specific interactions between MiP and prematurity, it is essential to understand the dysfunctions of regulatory immunity related to the risk of sepsis in a group of newborns affected by MiP and prematurity. We hypothesize that Treg might be impacted *in utero* by maternal or neonate risk factors and consequently associated with a risk of occurrence of sepsis during the first 72 hours of life. Our study aims to assess, on cord blood, the quantitative and functional characteristics of Treg subpopulations in neonates with EOS exposed to prenatal risk factors.

## Methods

### Study design and participants

Participants were delivered in 2 urban health centers (sub-urban-based group) and 3 University hospitals (hospital-based group) in the Abomey-Calavi, Sô-Ava, and Cotonou districts in the South Benin region where malaria is hyper-endemic (33). In both groups, only infants born from mothers living in the Abomey-Calavi district were recruited to facilitate the follow-up and minimize the effect of geographical origins. In the urban group, all consecutive births were included. In contrast, in the hospital group, only newborns delivered from mothers with maternal-fetal risk factors for infections (prematurity, prolonged rupture of the membrane, maternal fever) were included. In both groups, the exclusion criteria included HIV-positive status, major congenital malformation, and refusal of consent. All children from both groups had bi-monthly clinical follow-up visits at home or clinic during the first three months of life. Children were referred to the hospital to receive care whenever necessary for sickness, even for unscheduled emergency visits. The study protocol was approved by the local institutional review board (CER-ISBA 85 - 5 April 2016, extended on 3 February 2017) (34). Written informed consent was obtained from parents.

### Endpoints

The primary endpoint was clinical EOS diagnosis, and the secondary endpoint was mortality within the first three months of life. The hospital pediatrician established a neonatal sepsis diagnosis based on the child’s clinical examination and initial workup, including a haemogram, C-reactive protein (CRP), and microbiological cultures (blood, cerebrospinal fluid, and urine). EOS occurred within the first 72 hours following delivery (for a detailed algorithm for sepsis diagnosis, see the published study protocol (34)). Neonatal sepsis was suspected in neonates with perinatal infectious risk factors and more than two of the following criteria being present: neutrophil count <7500/mm3 or >14 500/mm3, band form >1500/mm3, immature/total neutrophils ratio >0.16, platelets count <150 000/mm3 and CRP >10 mg/L. Suspected neonatal sepsis was considered “clinical sepsis” when the following clinical signs were associated: temperature irregularity; respiratory distress or apnoea; seizures, altered tonus, irritability or lethargy; vomiting, altered feeding pattern, ileus; skin perfusion alteration, hemodynamic signs (tachycardia, hypotension); hypoglycemic/hyperglycemic, hyperlactatemia or identification of focal infection such as soft tissue infection or conjunctivitis. All newborns suspected of sepsis were subsequently adjudicated by two independent pediatricians and sorted into ‘presumed sepsis’ and ‘clinical sepsis.’ In addition to the entire medical file review, microbiological culture results were monitored during the adjudication. This analysis considered clinical EOS if confirmed by positive blood culture or adjudicated as clinical sepsis by both independent pediatricians. Presumed sepsis was considered if patients initially suspected for EOS and treated with antibiotics but not fulfilling criteria for clinical EOS.

At birth and follow-up visits, the clinical examination data of the children were collected. Blood samples as well as clinical data were obtained at birth, then at week (W)1, W4, W8 and W12. The study protocol has been described in detail elsewhere (34). MiP was defined as a malaria infection at delivery by positive placental or mother’s peripheral blood smear. The Lambaréné method was used to quantify parasitemia with a detection threshold of five parasites per microlitre (35). At delivery, the following maternal characteristics were collected: chorioamnionitis (only clinically diagnosed by the midwives when they observed amnionitis, vaginal or genital infection), fever at delivery (>38°C), abnormal amniotic fluid (fetid, meconial, hemorrhagic, yellow or tinged), premature rupture of membranes when >18 hours (PROM)), malaria at delivery, intermittent preventive treatment of malaria (IPT), age (years) and gravidity (**Tables 1**, **2**).

Table 1Maternal and infant characteristics between selected patients versus all cohort patients.

**Table:** 

	Phenotype done (n=98)	Phenotype not done (n=483)	All patients (n=581)	P-value*
Infant’s characteristics				
Female	53 (54.08)	234 (48.45)	287 (49.40)	0.321
Birth weight (grams)	2800 [2400–3100]	2843 [2396–3150]	2816 [2400–3150]	0.367
Low Birth weight < 2500 g	30 (30.61)	142 (29.40)	172 (29.60)	0.809
Prematurity	35 (35.71)	150 (31.06)	185 (31.84)	0.405
Gestational age (week)	38.0 [35.0–39.6]	38.5 [35.6–40.0]	38.4 [35.4–40.0]	0.245
< 37 weeks	26 (29.55)	124 (27.68)	150 (27.99)	0.391
≤ 32 weeks	8 (9.09)	26 (5.80)	34 (6.34)	
Haemoglobin (g/dL)	14.5 [13.2–16.2]	14.8 [13.3–16.3]	14.9 [13.3–16.3]	0.559
APGAR score at 1 minute	8 [7–9]	9 [8–9]	9 [8–9]	0.067
*Low < 7*	14 (14.29)	56 (11.62)	70 (12.07)	0.496
APGAR score at 5 minutes	10 [9–10]	10 [9–10]	10 [9–10]	0.662
*Low < 7*	5 (5.10)	24 (4.98)	29 (5.00)	1.000
Death	12 (12.24)	37 (7.66)	49 (8.43)	0.161

**Table:** 

Maternal characteristics	Phenotype done (n=89)	Phenotype not done (n=431)	All patients (n=520)	P-value*
Chorioamnionitis	11 (12.36)	43 (9.71)	54 (10.15)	0.444
Fever at Delivery (>38°C)	26 (29.21)	80 (18.06)	106 (19.92)	0.020
Abnormal amniotic fluid	52 (53.06)	247 (51.14)	299 (51.46)	0.741
PROM (>18 hours)	23 (25.84)	107 (24.15)	130 (24.44)	0.787
Malaria at delivery	30 (33.71)	13 (3.02)	43 (8.27)	<0.0001
IPT	73 (86.90)	398 (93.87)	471 (92.72)	0.036
Age (years)	26 [23–30]	26 [23–30]	26 [23–30]	0.491
Gravidity	2 [1–4]	3 [1–4]	3 [1–4]	0.213

Qualitative data are expressed as numbers and frequencies and quantitative data are expressed as medians and IQR (inter-quartile range: [Q1–Q3]). Qualitative variables were compared using the Chi-squared test (or Fisher’s exact test for small expected numbers). Quantitative variables were compared using the Wilcoxon-Mann-Whitney test. (statistically significant test *p ≤ 0.05*). PROM, Premature Rupture of Membranes; IPT, Intermittent Preventive Treatment of malaria; APGAR, neonatal adaptation score.

**Table 2:** Maternal and child’s characteristics according to the 4 groups analyzed.

	Non sepsis (n=53)		Sepsis (n=45)		All “phenotype” subjects (n=98)	P-value*
	Urban arm (n=12)	Hospital arm (n=41)	EOS Clinic (n=32)	EOS Presumed (n=13)		
Infant’s characteristics						
Female	8 (66.67)	24 (58.54)	14 (43.75)	7 (53.85)	53 (54.08)	0.507
Birth weight (grams)	3229 [3002–3544]	2800 [2500–3100]	2675 [1795–3030]	2400 [2200–2900]	2800 [2400–3100]	0.0007
Low Birth weight < 2500 g	0	10 (24.39)	13 (40.62)	7 (53.85)	30 (30.61)	0.008
Prematurity	0	14 (34.15)	15 (46.88)	6 (46.15)	35 (35.71)	0.015
Gestational age (week)	39.8 [39.3–40.5]	38.0 [35.2–39.3]	35.7 [32.3–39.8]	36.8 [34.6–38.1]	38.0 [35.0–39.6]	0.0132
< 37 weeks	0	13 (35.14)	8 (28.57)	5 (50.00)	26 (29.89	
≤ 32 weeks	0	1 (2.70)	7 (25.00)		8 (9.20)	
Haemoglobin (g/dL)	14.0 [12.8 -15.4]	14.8 [13.1–16.1]	14.3 [1.6–16.4]	14.9 [14.4–15.8]	14.5 [13.2–16.2]	0.3675
APGAR score at 1 minute	9 [8–9.5]	9 [8–9]	8 [6–8.5]	8 [6–9]	8 [7–9]	0.0027
*Low < 7*	0 (0.00)	1 (2.44)	10 (31.25)	3 (23.08)	14 (14.29)	0.001
APGAR score at 5 minutes	10 [10–10]	10 [9–10]	9 [8–10]	10 [8–10]	10 [9–10]	0.1473
*Low < 7*	0	1 (2.44)	4 (12.50)	0	5 (5.10)	0.254
Death	0	2 (4.88)	8 (25.00)	2 (15.38)	12 (12.24)	0.037
Cause of death		stillborn -hypoglycemia	sepsis	hypoglycemia		
Day of death		1	0 to 30	1 to 14		
Twins (Triplet)		2	4 (3)	3	9 (3)	
Single	12	39	25	10	86	
Maternal characteristics	12	39	27	11	89	
Chorioamnionitis	0	5 (12.82)	4 (14.81)	2 (18.18)	11 (12.36)	0.561
Fever at Delivery (>38°C)	1 (8.33)	10 (25.64)	12 (44.44)	3 (27.27)	26 (29.21)	0.131
Abnormal amniotic fluid	4 (33.33)	24 (61.54)	19 (70.37)	5 (45.45)	52 (58.43)	0.134
PROM (>18 hours)	0	8 (20.51)	10 (37.04)	5 (45.45)	23 (25.84)	0.022
Malaria at delivery	4 (33.33)	17 (43.59)	4 (14.81)	5 (45.45)	30 (33.71)	0.069
IPT	12 (100)	33 (89.19)	20 (76.92)	8 (88.89)	73 (86.90)	0.258
Age (years)	25 [23.5–29]	26 [23–31]	26 [23–30]	18 [25–30]	26 [23–30]	0.8736
Gravidity	2 [1.5–5]	3 [1–4]	2 [1–3]	2 [1–3]	2 [1–4]	0.8859

Qualitative data are expressed as numbers and frequency and quantitative data are expressed as medians and IQR (inter-quartile range: [Q1–Q3]). Qualitative variables were compared using the Chi-squared test (or Fisher’s exact test for small expected numbers). Anova test (or the Kruskal-Wallis test when distribution was not normal or when homoscedasticity was rejected) was performed to compare groups (statistically significant test *p ≤ 0.05*).

PROM, Premature Rupture of Membranes; IPT, Intermittent Preventive Treatment of malaria; APGAR, neonatal adaptation score.

**Figure 1:**
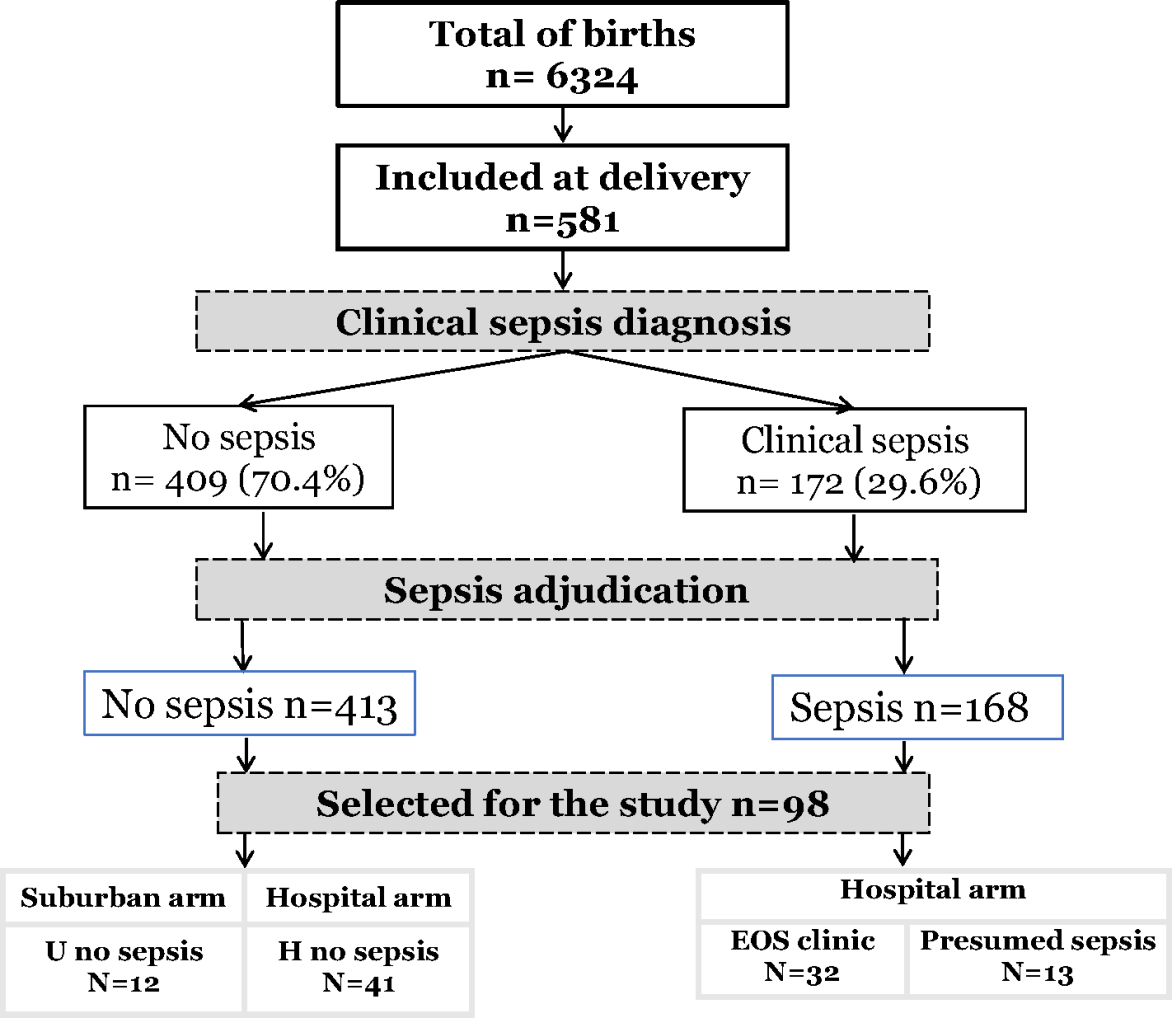
Flow-chart of study population.

For this study, among the 581 newborns included, 98 newborns from 89 mothers were selected with the most discriminating clinical profiles regarding clinical EOS, MiP, and prematurity occurrence. The newborns were divided into four groups: The urban non-sepsis group, regrouping healthy newborns with no prenatal risk factors (U no sepsis); the hospital group, all newborns with prenatal risk factors at delivery, subdivided into non-sepsis group (H no sepsis), clinical sepsis (EOS clinic) and presumed sepsis group (EOS presumed) (**Figure 1**).

### Cord blood collection

Cord blood (CB) was collected using a sterile technique. After the placenta was delivered, a section of the umbilical cord was cleaned with povidone-iodine or alcohol topical antiseptics. The umbilical vein was identified, and blood was obtained using a 21G needle. Blood was then collected into heparin-coated tubes, and samples were transported to the laboratory within 6 hours.

### Cell preparation and flow cytometry

Cord blood mononuclear cells (CBMCs) were isolated within 12 hours of collection by density-gradient centrifugation with Ficoll-Histopaque (Sigma, Darmstadt, Germany). Isolated CBMCs were gently resuspended in a cryopreservation medium containing 10% dimethyl sulfoxide (DMSO) and 90% fetal bovine serum (FBS) (Fisher Scientific, France). They were aliquoted into cryovials, stored at -80°C for 24 hours, and then transferred to a liquid nitrogen tank for subsequent analysis. For multicolor flow cytometry analysis, CBMCs were thawed and treated with human IgG (Miltenyi, France) to block Fc receptors and stained for surface markers with fluorochrome-labeled antibodies to characterize T-cell populations (**Supplementary Table 1**). After extracellular staining, cells were washed and fixed with Fixation/Permeabilization Buffer (BD Biosciences, USA) followed by Permeabilization Buffer (BD Biosciences, USA). Cells were finally stained for intracellular markers (**Supplementary Table 1**) and analyzed on FACS Canto-II (BD Biosciences). Two panels were defined to differentiate Treg cell subpopulations and their sources. For panel 1, cells were first stained with surface antibodies specific for CD3, CD4, CD25, CD127, CD45RA, CD31, CD39, and Fixable Viability Stain (FVS), followed by intranuclear staining for CTLA-4 and FOXP3. For Panel 2, cells were first stained with surface antibodies, including anti-CD3, anti-CD4, anti-CD25, anti-CD127, anti-CD49d, anti-CD279 (PD1), anti-Integrinß7, and FVS, followed by intranuclear staining with anti-FOXP3. Fluorescence Minus One (FMO) control was used to identify any background spread of fluorochromes and to establish gating boundaries. Live cells were gated based on forward- and side-scatter properties and the absence of fluorescence in the FVS cells kit (BD Biosciences, USA). Samples with viability of more than 90% were considered for analysis. Flow cytometry analysis was performed using FACSDiva software version 8 (BD Biosciences). Compensation for 10 and 9 color stain sets was accomplished using either (CompBeads, BD) or CBMCs stained for viability. Then, single stained cells with different mAbs used in the study were run to ensure that each stain was the brightest in its channel before acquisition. Manual compensation was used, allowing for an optimal adjustment of the spectral overlap between the different fluorochromes. FlowJo software version 10.8.1 was used to analyze data from flow cytometry acquisition. The gating strategies are presented in **Supplementary Figures 1** and **2**.

### RNA extraction, reverse transcription, and quantitative PCR

Total RNA was extracted from cord blood by QIAsymphony SP/AS (QIAGEN Hilden, Germany) using PAXgeneTM Blood RNA Kit (PreAnalytix, Hilden, Germany) according to manufacturer guidelines. The RNA integrity was measured before RNA amplification using a Bioanalyser 2100 (Agilent Technologies, Palo Alto, CA) by the manufacturer’s instructions (RNA integrity number ≤ 6 were excluded [min 6.5–max 9.4]). The expression level of CX3CR1 and CD74 was measured by RT-qPCR using an ABI7500 thermocycler (Applied BioSystems., California, USA) from 10 ng of RNA samples using prototype Argene—kit (bioMerieux, France) following the manufacturer’s instructions. The copy concentration of each gene is determined using a calibration curve. The results are expressed as the ratio of the concentrations of CX3CR1 or CD74 to those of HPRT1 (Hypoxanthine Phosphoribosyltransferase 1) used to normalize the gene expression results.

### Protein quantification

PCT, IL-6, IL-10, and IP-10 proteins in plasma were analyzed in a batch by Simplex AssaysTM according to the manufacturer’s instructions as previously described (36). The Ella microfluidic analyzer (Protein Simple, San Jose, CA, USA) was used to assess cytokine concentrations (37).

### Statistical analyses

Nominal variables are reported as frequencies and continuous variables are reported as medians ± standard deviations (SD) (parametric data) or as medians (25^th^–75^th^ percentiles) (nonparametric data). Normality testing was performed using the Kolmogorov-Smirnov test. Data were analyzed using Stata statistical software (Stata/IC for Mac, version 16, TX, USA) and GraphPad Prism 9 (GraphPad Software, La Jolla, CA, USA). Nominal variables were compared using chi-square analysis or Fischer’s exact test as appropriate. Group medians were compared using Mann-Whitney U or Kruskal-Wallis tests. Group means were compared using an independent samples t-test or ANOVA. Spearman rank or Pearson’s correlation tests were used to measure the strength of association between variables. A p-value ≤0.05 was considered statistically significant.

We used univariate and multivariate logistic regression models to study clinical items and biomarker factors associated with clinical sepsis. We performed a model with three modalities (EOS clinic/Presumed sepsis/no sepsis). All variables with a p-value below 0.05 in univariate analysis were selected for the multivariate analysis. In addition, all biomarkers were forced into the multivariate models. Due to the collinearity between the frequency of Treg subpopulations and their different marker expression, we only included the frequencies of the different Treg subpopulations in the multivariate model. Then, a manual backward selection procedure was used to obtain the final adjusted multivariate model; a p-value of <0.05 was considered statistically significant. Stata version 16 for Macs (Stata/IC for Mac, version 16, TX, USA) was used for statistical analyses.

## Results

### Patient characteristics

Of the 6324 births occurring during the study period, 581 newborns were recruited. Pediatricians identified 172 infants with suspected sepsis (172/581, 29.6%). After adjudication, diagnosis of sepsis was maintained in 168 patients (168/172, 97.6%), most having early onset sepsis (163/168, 97,0%). (**Figure 1**). Among all, a sample of 98 patients (98/581, 16.9%) was selected and subdivided into four groups of analysis: Healthy urban (U no sepsis, n=12), healthy hospital (H no sepsis, n=41), clinical sepsis (clinical EOS, n=32), and presumed sepsis (EOS presumed, n=13). Among clinical sepsis group, positive blood culture occurred in 16 patients with identification of mainly*, E. coli, Enterobacter spp, Serratia marcescens, Klebsiella pneumoniae, Moraxella sp, and Staphylococcus*. Maternal and newborn characteristics between patients selected for this study and the whole cohort are displayed in **Table 1**. Within the 98 newborn, we had 8 twins and 1 triplet. For newborn traits, there were no statistically significant differences between the study group and the non-study group for gender, birth weight, prematurity (gestational age < 37 weeks), hemoglobin, and APGAR score at 1 and 5 minutes. The majority of the deaths occur within the first week of life and were directly associated to EOS. Regarding maternal characteristics, there were no statistically significant differences between the study group and all patients for maternal age, gravidity, chorioamnionitis, abnormal amniotic fluid, and prolonged membrane rupture (PROM) >18 hours. Because of the selection of neonates with the most discriminating clinical profiles, frequencies of maternal malaria infection (MiP) and fever at delivery were significantly higher in the study group compared to the non-study group (respectively: p<0.0001, p<0.020). Among the studied patients, maternal and newborn characteristics were compared in the four studied groups (**Table 2**). Low birth weight, prematurity, Low APGAR score < 7 at 1 minute, Maternal fever at delivery, and PROM are more dominant in the EOS clinic group.

### Reduced frequency of Treg, effectors T cells, and higher frequency of activated Treg in neonates with clinical sepsis

**Figure 2:**
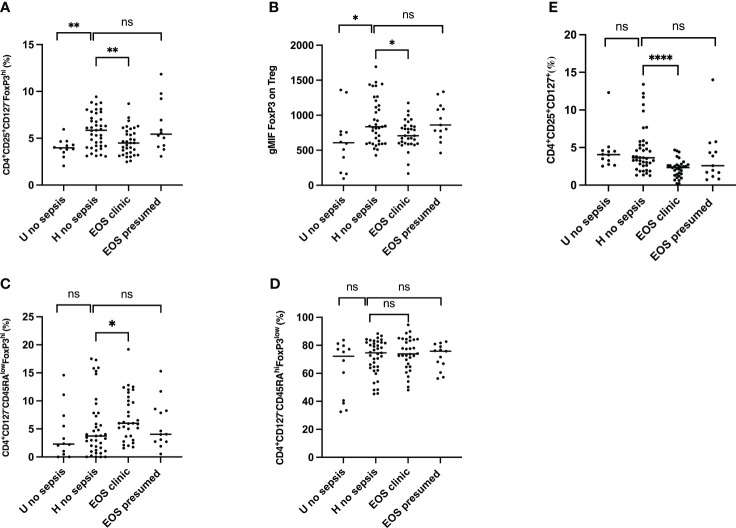
Frequencies of regulatory T cells (Treg), activated Treg, effector T cells and FoxP3 expression cells from the cord blood of subgroups of study cohort. **(A)** Frequencies of CD4^+^CD25^+^CD127^-^FoxP3^+^ regulatory T cells. **(B)** FOXP3 expression on Treg. geometric Mean Fluorescence Intensity (gMFI) of Forkhead box P3 (FoxP3) in CD4^+^CD25^+^CD127^-^FoxP3^+^ regulatory T cells is shown. **(C)** Frequencies of CD4^+^CD127^-^CD45RA^low^FoxP3^hi^ activated Treg cells are shown. **(D)** Frequencies of CD4^+^CD127^-^CD45RA^hi^FoxP3^hi^ resting Treg cells are shown. **(E)** Frequencies of CD4^+^CD25^+^CD127^+^ effector T cells are shown. EOS neonates (clinic (n=32) and presumed (n=13)) are compared to Hospital non sepsis neonates (n=41), as controls. Hospital non sepsis neonates is compared to sub-urban non sepsis neonates (n=12) to evaluate risk factors impact. The plots are shown with median and minimum/maximum values. p-values were calculated by the Mann–Whitney U-test. *P*<0.05 indicates a statistically significant difference. *p* values: *p<0.05, **p<0.01, ***p<0.001, ****p<0.0001. ns, no statistically significant difference.

We first observed that circulating Treg frequencies (CD3^+^CD4^+^CD25^+^CD127^-^FOXP3^+^) and expression of Foxp3 in CD3^+^CD4^+^CD25^+^CD127^-^ cells were significantly increased in patients with prenatal risk factors but were not seen if associated with clinical sepsis (**Figures 2A, B**). Patients with presumed sepsis failed to show a decrease in circulating Treg frequency and Foxp3 expression compared to healthy patients with prenatal risk factors, as seen with clinical sepsis. Different Treg cells subpopulations were then analyzed (resting: CD3^+^CD4^+^CD25^+^CD127^-^CD45RA^high^FoxP3^low^; activated: CD3^+^CD4^+^CD25^+^CD127^-^CD45RA^low^FoxP3^high^ (38) and effector T cells CD3^+^CD4^+^CD25^+^CD127^+^). While resting Treg frequency remains constant across all groups (**Figure 2D**), activated Treg frequencies were significantly higher in neonates with clinical sepsis (**Figure 2C**). The proportion of Effectors T cells was significantly lower in neonates with clinical sepsis compared to healthy neonates with prenatal risk factors. Still, no difference was seen between the latter and newborn with presumed sepsis, confirming that patients with presumed sepsis had a significantly different T cell phenotype than those with proven clinical sepsis (**Figure 2E**). This suggests that, on cord blood analysis, prenatal risk factors trigger a specific T cell phenotype, consisting of an increase in T regulatory cells, without an increase in effector, activated, and resting cells. This phenotype is similarly seen in patients with prenatal risk factors and presumed sepsis but is modified in patients who will subsequently develop clinical sepsis with a decrease in Treg and T effector frequency, FoxP3 expression, but an increase in activated T cells. We hypothesize that decreasing Treg cells in patients developing sepsis compared to patients with risk factors without sepsis might be due to either a migration of cells, such as T cell homing, or an innate or acquired defect in response to risk factors. However, the presence of activated T cells in sepsis may not support the hypothesis of a defective reaction, especially regarding the T cell phenotypes in patients with presumed sepsis.

### Increasing frequency and expression of CTLA-4, PD-1, and CD39 in Treg pool in neonates with sepsis

**Figure 3:**
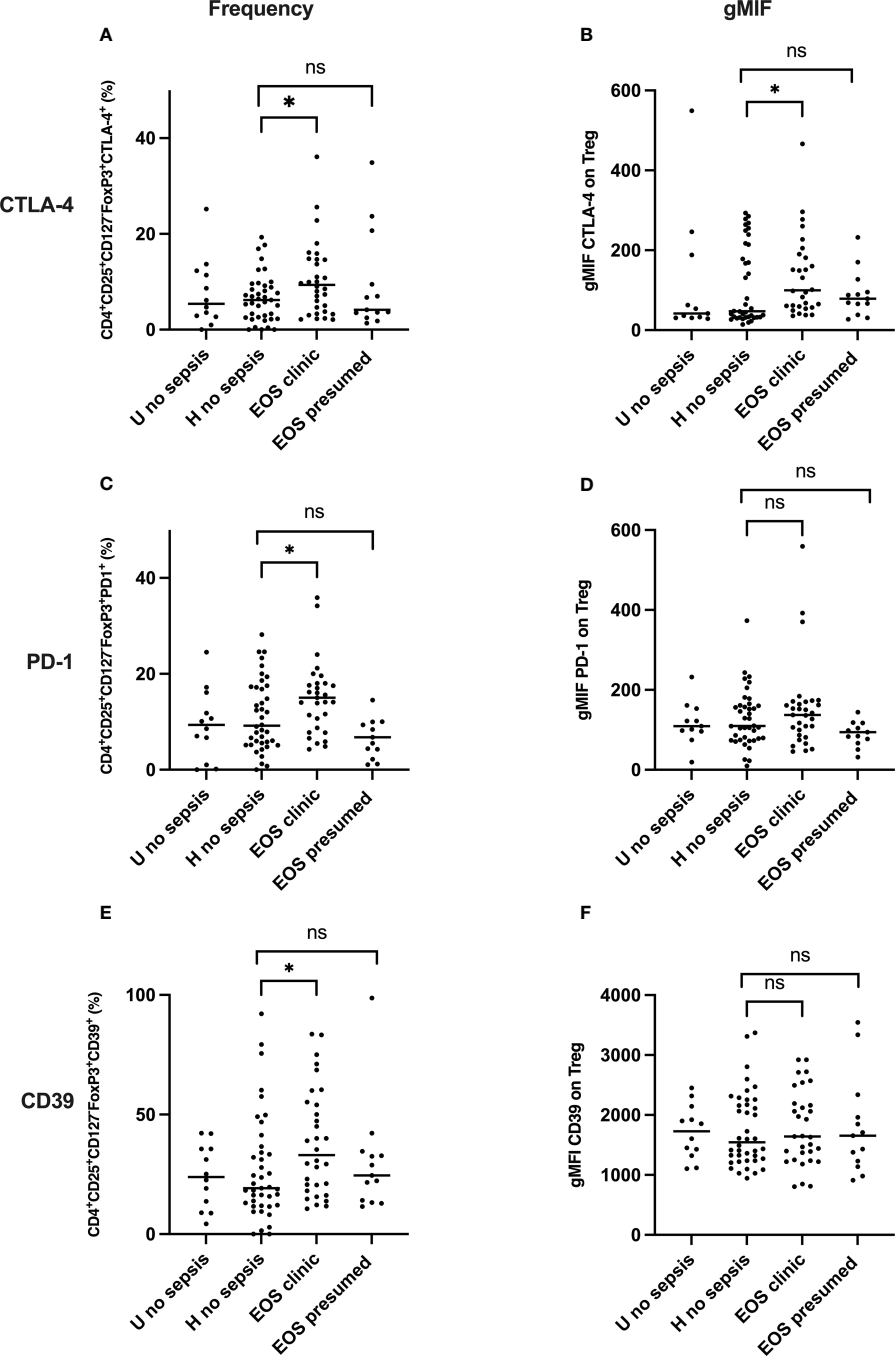
Frequency and expression of CTLA-4, PD-1 and CD39 on Treg pool from the cord blood of subgroups of study cohort. **(A, C, E)** dot plots compare the frequencies of Treg CTLA-4^+^, Treg PD-1^+^, Tregs CD39^+^respectively between neonates’ subgroups: EOS clinic (n=32), EOS presumed (n=13), Hospital no sepsis (n=41), sub-urban no sepsis (n=12). **(B, D, F)** representative dot plots of the comparison of CTLA-4, PD-1, CD39 expression level on Treg surface between neonates’ subgroups. EOS neonates (clinic and presumed) are compared to Hospital non sepsis neonates, as controls. The plots are shown with median and minimum/maximum values. p-values were calculated by the Mann–Whitney U-test. *P*<0.05 indicates a statistically significant difference. *p* values: *p<0.05, **p<0.01, ***p<0.001, ****p<0.0001. ns, no statistically significant difference.

Knowing that CTLA-4, PD-1, and CD39 are expressed in Treg but only upregulated following T cell activation (39–41), we analyzed the frequency of CTLA-4 on Treg. As shown in **Figures 3A, B**, CTLA-4^+^ Treg (CD3^+^CD4^+^CD25^+^CD127^-^FOXP3^+^CTLA-4^+^) frequencies and CTLA-4 expression were significantly higher in neonates with clinical sepsis compared to healthy subjects with prenatal risk factors. CTLA-4^+^ Treg frequencies were not different among healthy newborns (with or without perinatal risk factors) and neonates with presumed sepsis. In the same way, the co-stimulatory molecules PD-1 and exhaustion markers CD39 frequency (respectively, CD3^+^CD4^+^CD25^+^CD127^-^FOXP3^+^ cells and CD3^+^CD4^+^CD25^+^CD127^-^FOXP3^+^PD-1^+^) (**Figures 3C, E**), showed an increase in neonates developing sepsis compared to healthy newborn and those with presumed sepsis. In contrast, PD-1 and CD39 expression on Treg cells were similar between groups (**Figures 3D, F**). This confirms that Treg activation i) is signing perinatal events associated with neonatal EOS and ii) is independent of prenatal risk factors. As such, this suggests that neonatal sepsis is driving a specific and oriented response on T cells, and a decrease in Treg frequency might be related to the extravascular migration of T cells.

### Increased frequencies of the homing integrin α4β1 Treg in neonatal sepsis

**Figure 4:**
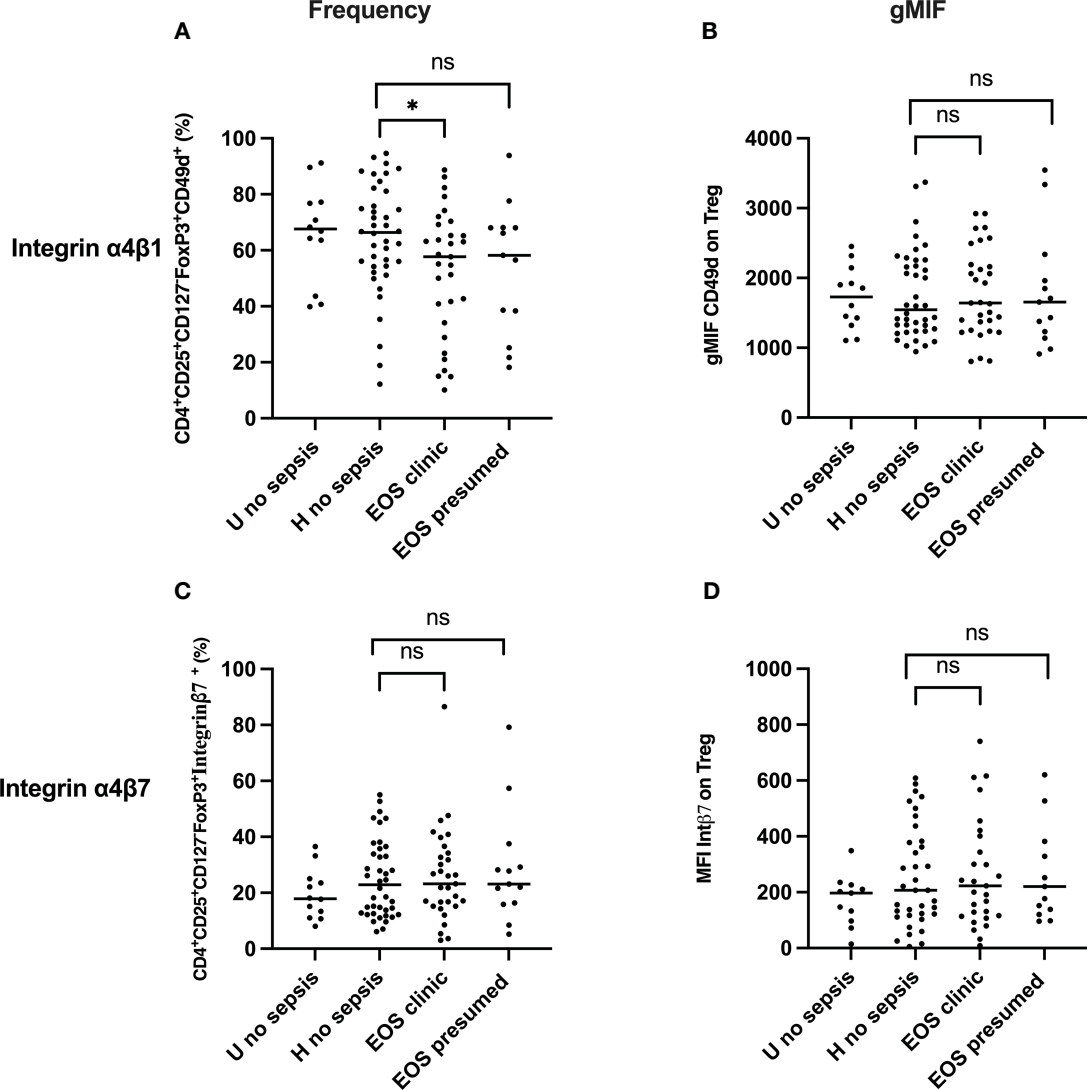
Frequency and expression of homing molecules (Integrin α4β1 & Integrin α4β7) Treg. **(A, C)** Frequency of Treg Integrin α4β1^+^ and Treg Integrin α4β7^+^cells are shown. **(B, D)** Integrin α4β1 and Integrin α4β7 expression on Treg. EOS clinic (n=32), EOS presumed (n=13), Hospital no sepsis (n=41), sub-urban no sepsis (n=12). geometric Mean Fluorescence Intensity (gMFI) of Integrin α4β1 and Integrin α4β7 in CD4^+^CD25^+^CD127^-^FoxP3^+^ regulatory T cells is shown. EOS neonates (clinic and presumed) are compared to Hospital non sepsis neonates, as controls. The plots are shown with median and minimum/maximum values. p-values were calculated by the Mann–Whitney U-test. *P*<0.05 indicates a statistically significant difference. *p* values: *p<0.05, **p<0.01, ***p<0.001, ****p<0.0001. ns, no statistically significant difference.

Integrin α4β1 and integrin β7 are usually used as homing markers of Treg cells, respectively, tissue homing and gut homing (42, 43). Thus, we further assessed these homing molecules’ frequency and expression on circulating Treg cells in cord blood. The frequency of integrin α4β1 and α4β7 Treg were analyzed, and levels of expression of both integrins were measured on Treg cells. As shown in **Figure 4A**, Integrin α4β1^+^ Treg (CD3^+^CD4^+^CD25^+^CD127^-^FOXP3^+^Integrinα4β1^+^) frequencies were significantly lower in patients developing neonatal sepsis compared to healthy subjects with prenatal risk factors. In contrast, the frequency of integrin α4β7+ Treg (CD3+CD4+CD25+CD127-FOXP3+Integrinβ7+) was similar among all groups. Expression on Treg cells of integrin α4β1 and integrin α4β7 on Treg were not significantly different among groups (**Figures 4B, D**). In addition, the expression of integrin α4β1 and integrin α4β7 on CD4^+^CD25^+^CD127^+^ effector T cells was not significantly different among the groups, as shown in **Supplementary Figure 4**. This confirms that Treg cell homing occurs preferentially in the endothelium than in the gastrointestinal tissue compartment. In addition to the homing, the origin of Treg in both patients with prenatal risk factors and those with neonatal sepsis is questioned.

### Reduced recent thymic emigrant and increase of peripherally induced Treg in newborns with sepsis

**Figure 5:**
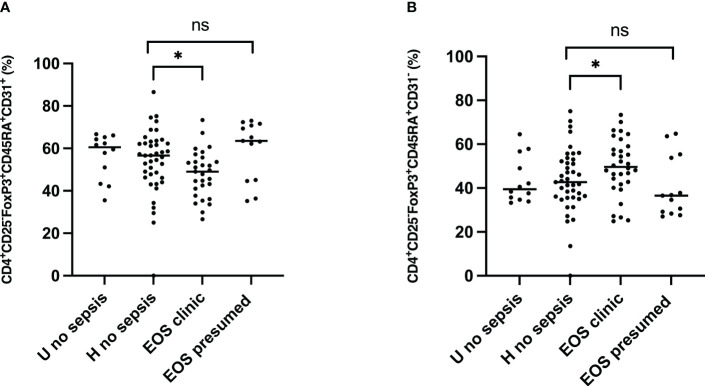
Origin of Treg subpopulations in neonates. Tregs origin were examined for expression of the surface markers CD45RA and CD31. EOS clinic (n=32), EOS presumed (n=13), Hospital no sepsis (n=41), sub-urban no sepsis (n=12). **(A)** Representative dot plot comparing median percentage of CD3^+^CD4^+^CD25^+^FOXP3^+^CD45RA^+^CD31^+^ Recent thymic emigrant Treg from the cord blood of subgroups of study cohort. **(B)** Representative dot plot comparing median percentage of CD3^+^CD4^+^CD25^+^FOXP3^+^CD45RA^+^CD31^-^ Peripherally induced naïve Treg from the cord blood of subgroups of study cohort. EOS neonates (clinic and presumed) are compared to Hospital non sepsis neonates, as controls. The plots are shown with median and minimum/maximum values. p-values were calculated by the Mann–Whitney U-test. *P*<0.05 indicates a statistically significant difference. *p* values: *p<0.05, **p<0.01, ***p<0.001, ****p<0.0001. ns, no statistically significant difference.

The surface expression of CD45RA and CD31 identified the Treg subpopulation’s origin. The positive surface expression of CD45RA and CD31 defined recent thymic emigrant (RTE) Treg. The percentage of peripherally induced naïve regulatory T cells (CD3^+^CD4^+^CD25^+^FOXP3^+^CD45RA^+^ CD31^-^) (**Figures 5A, B**) was significantly higher in neonates developing sepsis compared to another group. At the same time, the frequency of RTE Treg (CD3^+^CD4^+^CD25^+^FOXP3^+^ CD45RA^+^CD31^+^) was considerably lower, suggesting that Treg cells are more peripherally induced in newborns developing sepsis. The effect of prenatal risk factors is not apparent.

### Impact of Treg activation and homing on neonatal sepsis prognosis and diagnosis

**Figure 6:**
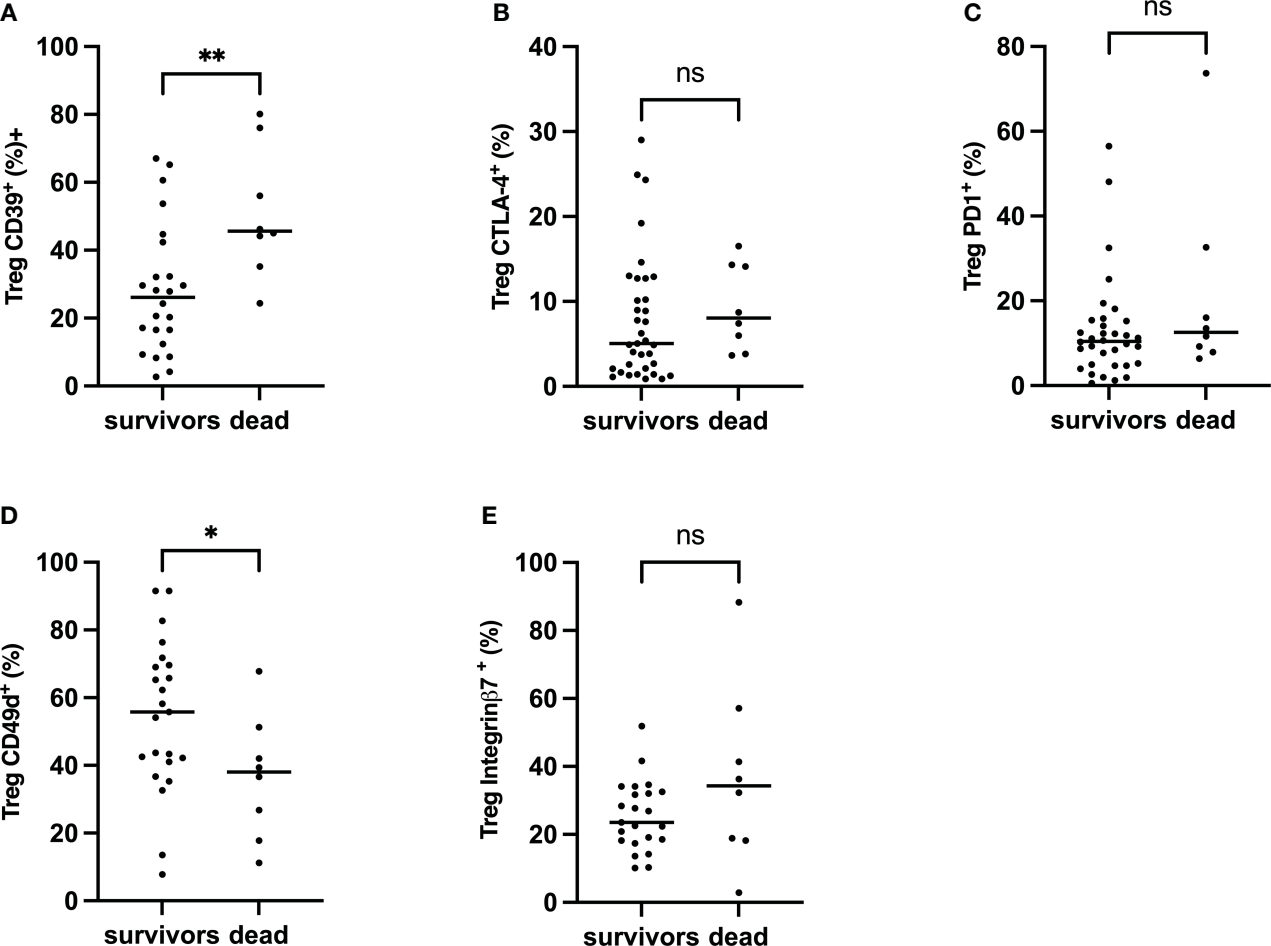
Frequency of CTLA-4, PD-1, CD39 α4β1 and α4β7 on Treg, Comparison between survivors and non-survivors in EOS neonates. Mann–Whitney U-test was performed for comparison. **(A)** Percentage of Treg CD39^+^. **(B)** Percentage of Tregs CTLA-4^+^. **(C)** Percentage of Tregs PD-1^+^. **(D)** Percentage of Treg Integrin α4β1^+^. **(E)** The percentage of Treg Integrin α4β7^+^. The plots are shown with median and minimum/maximum values. p-values were calculated by the Mann–Whitney U-test. *P*<0.05 indicates a statistically significant difference. *p* values: *p<0.05, **p<0.01, ***p<0.001, ****p<0.0001. ns, no statistically significant difference.

Considering the burden of neonatal sepsis on the outcome, the impact of Treg activation and homing markers seen in newborns may be associated with prognosis. First, CTLA-4, PD-1, CD39, Integrin α4β1, and integrin α4β7 on Treg were assessed in patients with sepsis. CD39^+^Treg cells were significantly increased in deceased patients compared to sepsis survivors, whereas no difference was seen with CTLA-4 and PD1. Integrin α4β1 Treg was reduced considerably in deceased newborns when there was no difference in the Integrin α4β7 Treg pool (**Figure 6**). This suggests that CD39 and Integrin α4β1, signing Treg exhaustement and endothelial homing, may be associated with neonatal sepsis prognosis.

A multivariate analysis of prenatal risk factors associated with sepsis was performed to evaluate the relative impact and independence of cord blood Treg cell phenotypic signature on neonatal sepsis diagnosis (**Table 3**). All Treg markers, but CD39 and CD45RA^+^CD31^-^ were independently associated with clinical sepsis and not presumed sepsis, suggesting that the main phenotypic Treg characteristics identified in this study, such as Treg frequency, T effector frequency, Integrin α4β1and activation markers CTLA-4 and PD1 be associated with the development of neonatal sepsis.

**Table 3:** Logistic regression analysis of the risk factors for neonatal sepsis.

Characteristics	Univariate		Multivariate	
	_crude_ OR [95% CI]	p value	_Adjusted_ OR [95% CI]	p value
EOS Clinic				
Effectors T cells (%)	0.54 [0.37; 0.78]	0.001	0.13 [0.03; 0.46]	0.001
Treg (%)	0.98 [0.95; 1.00]	0.097	0.92 [0.86; 1.00]	0.024
Treg CD39^+^ (%)	1.02 [1.00; 1.04]	0.040	0.97 [0.91; 1.03]	0.388
Treg CD49d^+^ (%)	0.97 [0.95; 1.00]	0.023	0.92 [0.87; 0.98]	0.008
Treg CTLA-4^+^ (%)	1.02 [0.98; 1.06]	0.261	1.11 [1.00; 1.23]	0.046
Treg PD-1^+^ (%)	1.05 [1.00; 1.14]	0.065	1.16 [1.00; 1.34]	0.046
Peripherally induced Treg (%)	1.03 [0.99; 1.07]	0.061	1.04 [0.96; 1.15]	0.287
Preterm birth	1.76 [1.11; 2.81]	0.016	13.7 [1.40; 135]	0.024
Maternal infection	3.69 [1.89; 7.18]	< 0.001	0.11 [0.04; 3.01]	0.195
Maternal fever	3.33 [2.03; 5.46]	< 0.001	43.4 [3.36; 559]	0.004
Abnormal amniotic fluid	0.89 [0.56; 1.41]	0.627	–	–
PROM	1.69 [1.03; 2.77]	0.037	0.11 [0; 2.61]	0.174
Apgar at 1 min *<* 7	3.18 [1.74; 5.81]	< 0.001	–	–
Malaria at delivery	0.75 [0.34; 1.66]	0.491	0.05 [0; 0.78]	0.033
Gravidity	1.27 [0.76; 2.13]	0.352	–	–
Low birthweight	1.70 [1.07; 2.72]	0.025	–	–
Sex (Female)	0.87 [0.55; 1.37]	0.554	–	–
IPT	2.10 [1.05; 4.19]	0.034	–	–
EOS presumed				
Effectors T cells (%)	0.48 [0.31; 0.74]	0.400	0.84 [0.62; 1.15]	0.297
Treg (%)	1.00 [0.97; 1.00]	0.739	1.00 [0.95; 1.05]	0.904
Treg CD39^+^ (%)	1.02 [1.00; 1.04]	0.633	0.99 [0.95; 1.04]	0.881
Treg CD49d^+^ (%)	0.97 [0.95; 1.00]	0.024	0.99 [0.91; 1.00]	0.037
Treg CTLA-4^+^ (%)	1.00 [0.95; 1.06]	0.854	1.02 [0.96; 1.08]	0.461
Treg PD-1^+^ (%)	1.06 [1.00; 1.14]	0.039	1.00 [0.90; 1.11]	0.935
Peripherally induced Treg (%)	1.03 [0.94; 1.03]	0.514	0.99 [0.93; 1.05]	0.835
Preterm birth	1.83 [1.03; 3.24]	0.038	4.45 [0.89; 31]	0.066
Maternal infection	3.31 [1.49; 7.34]	0.003	0.46 [0.04; 5.28]	0.537
Maternal fever	3.33 [0.85; 3.11]	0.134	1.5 [0.17; 8.90]	0.650
Abnormal amniotic fluid	1.14 [0.63; 2.05]	0.653	–	–
PROM	2.41 [1.34; 4.34]	0.003	3.46 [0.51; 23]	0.201
Apgar at 1 min *<* 7	2.82 [1.36; 5.85]	0.005	–	–
Malaria at delivery	0.90 [0.35; 2.29]	0.835	1.64 [0.31; 8.66]	0.556
Gravidity	0.70 [0.39; 1.27]	0.253	–	–
Low birth weight	2.00 [1.12; 3.55]	0.018	–	–
Sex (Female)	1.05 [0.59; 1.85]	0.863	–	–
IPT	1.91 [1.05; 4.19]	0.147	–	–

To evaluate the accuracy of new diagnostic and prognostic biomarkers for neonatal sepsis targeting the host response in our selected study subjects, certain transcriptional biomarkers (CD74, CX3CR1) and protein biomarkers (PCT, IL-6, IL-10, IP-10) were also analyzed, as in our previous study on the same cohort (44). Only IL-6 was significantly higher in the EOS clinical group (**Supplementary Figure 3**, **Supplementary Table 2**).

## Discussion

Hereby, we demonstrated that 1) prenatal risk factors induce specific Treg response with an increase in Treg cells without activation and homing, 2) newborn developing sepsis present at birth a T cell signature with activation, endothelial homing, and exhaustement of Treg cells from mostly peripherally induced Treg cells, 3) this signature is independently associated with a forthcoming sepsis diagnosis as well as sepsis prognosis.

Prenatal risk factors are known to be associated with an increase in Treg cell frequency. Low gestational age was inversely associated with Treg frequency, with premature infants having increased Treg cell frequency compared to full-term infants and adults (45, 46). Other perinatal risk factors are associated with Treg increases in newborns. Brustoski et al. observed a higher percentage of circulating Treg in the cord blood of neonates born to mothers infected with *P. falciparum* compared to those born to uninfected mothers, suggesting that the fetal immune status might be modified *in utero* by exposure to MiP (47). One factor that may influence Treg induction is the timing of exposure to malarial antigens during fetal immune development. Prahl et al. found that *in-utero* exposure to malaria antigen during the first or early second trimester is more likely to induce Treg differentiation, whereas infections occurring closer to term may not have the same effect (48). Another study showed that the frequency of Treg was significantly higher in the CBMCs of Kenyan newborns born to mothers infected with *P. falciparum* than in the CBMCs of North American newborns (49). We also confirmed the increase in Treg, unactivated cell frequency in newborns born with prenatal risk factors, including MiP.

Few studies evaluated Treg response during sepsis. Pagel et al. demonstrated that, on day 3, the frequency of Treg was higher in premature infants with EOS, regardless of gestational age (46). Interestingly, an increase in Treg frequency was shown in adults with sepsis-associated mortality (50). Treg depletion is reported to be associated with decreased mortality (50, 51). These observations contrast with our results, which show a reduced frequency of Treg and expression of FoxP3 in newborns developing EOS compared to healthy ones with perinatal risk factors. Similarly, Fortmann et al. showed that newborns with LOS showed a decrease in CD4^+^FoxP3^+^ T cells compared to controls (52). Treg frequency might be associated with or predispose to neonatal sepsis, which is probable. The logistic regression model shows that the decreased frequency of Treg cells in cord blood is independently associated with the risk of occurrence of clinical EOS and conversely that an increase in these cells at birth would protect against the risk of sepsis. In a context where the dynamic of Treg cell modifications is subject to conflicting results, the sepsis phase and sampling timing may influence its kinetics. Our study evaluated specifically Treg signature at delivery on cord blood. Our data further illustrate that other Treg cell subpopulations (38) discriminate newborns who develop sepsis from those without sepsis, but they are associated or not with prenatal risk factors. Our results are consistent with those of Timperi et al., who noted a high frequency of activated Treg cells in neonates affected by sepsis and showed an inverse correlation between the frequency of activated Treg cells and the severity of the clinical sepsis (53). In addition to the reduced frequency of the Treg pool, we showed that effector T cells were concomitantly lower in patients developing sepsis, suggesting radical modifications of most Treg subpopulations.

Among the different hypotheses explaining the decrease of circulating Treg in newborns developing sepsis, extravascular migration may be explanatory. This hypothesis is indirectly supported by multiple reports showing that homing of Treg occurs as gestational age increases and continues during the first 18 months of life, preferentially to the gastrointestinal compartment (54, 55). With this, we measured Treg subsets with migration markers such as integrins α4β1 and α4β7. Integrin α4β1 is the vascular cell adhesion molecule (VCAM-1) present on endothelial cells and enables cell migration to diverse sites, including the genital tract, intestine, lungs, and brain. Integrin α4β7 is essential for migration to gut-associated lymphoid tissues by interaction with its ligand MAdCAM-1 (mucosal addressing cell adhesion molecule-1) (42, 43). We showed a decreased frequency of α4β1^+^ Treg in the cord blood of newborns who will develop clinical EOS and identical frequencies of α4β7^+^ Treg in all newborns, suggesting that the Treg cells have migrated to endothelium at the sites infections and as in the gut as commonly occurring in healthy newborns (55).

The role of CTLA-4 and PD-1 are important inhibitory molecules in antigen-specific T-cell activation. In the cord blood of newborns developing sepsis, we observed higher frequencies of CTLA-4^+^ and PD-1^+^ Treg. Conversely, these newborns are born with a lower frequency of effector T cells. Those higher frequencies of Treg CTLA-4^+^, Treg PD-1^+^, and the low frequency of Effector lymphocytes are independently associated with the risk of occurrence of clinical EOS. Previous studies have shown that higher expression levels of CTLA-4 and PD-1 on CD4^+^ T lymphocytes, CD8^+^ cells, and Treg were observed in the spleen of septic mice compared to controls (56, 57). These changes are accompanied by apoptosis, death, and exhaustion of T cells (56, 57). Clinical studies also suggest that elevated PD-1 expression on T cells in patients with sepsis is significantly associated with decreased T cell proliferation and increased secondary nosocomial infection (28, 58).

Additionally, increased expression of CTLA-4 on T cells is associated with T cell depletion, resulting in an immunocompromised state in patients (59). Therefore, the inhibitory molecules PD-1 and CTLA-4 play a crucial role in the immunosuppressive phase of sepsis. They may participate in immune regulation in sepsis by negatively regulating T cell function (60, 61). Like Wang et al., we observed high frequencies of CTLA-4^+^ Treg and PD-1^+^ Treg in the cord blood of EOS patients (58, 62). Altogether, our results suggest that these high frequencies of CTLA-4^+^ Treg and PD-1^+^ Treg, signing Treg activation, negatively regulated the proliferation and activation of T cells supported by the low frequency of effector T cells observed, therefore participating in inhibiting the ability of neonate EOS immune responses to eliminate pathogens.

Interestingly, our results showed that Treg activation is independent of the presence of prenatal risk factors. Through the metabolization of extracellular ATP to adenosine, CD39^+^ has been shown to play a central role in the induction of immunosuppressive activity on immune cells with decreased CD39 expression signing Treg exhaustment (63). Huang et al. showed that the circulating level of CD39^+^ Treg increased significantly in sepsis patients compared to patients with non-septic inflammation patients and healthy control subjects, suggesting that the frequency or MFI of CD39^+^ Treg may serve as a biomarker to predict the outcome of sepsis (29). In our study, newborns developing clinical EOS are already born with a high frequency of CD39^+^ Treg in the cord blood, suggesting an early diagnostic signature. In addition, the frequency of CD39^+^ Treg was significantly higher in the deceased compared to survivor patients with EOS. Our data prove the association between CD39^+^ Treg frequency, sepsis development, and its outcome. Prognosis of EOS was also independently associated with lower α4β1^+^Treg frequency, signing Treg homing.

We analyzed the two physiologic origins of Treg in the cord blood, namely Treg recently emigrated from the thymus (RTE) and Treg induced at the periphery. Luciano et al. showed that the frequency of Treg induced at the periphery was significantly higher in the cord blood of premature infants associated with inflammation and prenatal exposure to Gram-negative bacteria and suggested that a change in the homeostatic composition of Treg subsets related to a reduction in *de novo* generation of Treg cells related to prematurity and exposure to chorioamnionitis and circulating endotoxins during the pregnancy (45). Similarly, our study describes a preponderance of the peripherally induced Treg over the RTE Treg pool in children who will develop clinical EOS. This may be due to a decrease in the efficiency of the thymus to produce RTE Treg or to a more significant induction of peripherally induced Treg via an infective process initiated before birth, prematurity, or other prenatal risk factor (45).

The clinical pertinence of the detailed phenotypic characterization of Treg cells in newborns was assessed through a multivariable analysis to identify their independence from the EOS diagnosis. The multivariate logistic regression model confirms that prematurity and maternal fever are independently associated with the risk of EOS clinic and conversely that MiP would protect against EOS. It further confirms the independent association of decreased Treg cell frequency, activation, and homing with EOS, suggesting that Treg, Integrin α4β1, activation markers CTLA-4, PD-1 and CD39 may become robust early markers of neonatal sepsis.

In conclusion, the present study depthfully investigates Treg signature in various clinical conditions representative of real life, healthy term newborns without prenatal risk factors, healthy newborns with prenatal risk factors, newborns with prenatal risk factors and suspected sepsis, and newborns with clinical early onset sepsis. However, prenatal risk factors for sepsis were associated with a specific Treg response signed by an increased frequency of unactivated Treg cells without homing; newborns developing sepsis show reduced Treg frequency, activation, and homing, most probably to the endothelium. This study needs to be confirmed, but this signature’s robustness may suggest that diagnosis of EOS neonatal sepsis could be identified at delivery before clinical sepsis occurred. This study highlights the potential role of Treg cell dynamics in the pathogenesis of neonatal sepsis and underscores the importance of further research in this area.

## Data availability statement

The raw data supporting the conclusions of this article will be made available by the authors, without undue reservation.

## Ethics statement

The studies involving humans were approved by L’institut des sciences biomédicales appliquéesCER-ISBA 85-5a. The studies were conducted in accordance with the local legislation and institutional requirements. Written informed consent for participation in this study was provided by the participants’ legal guardians/next of kin.

## Author contributions

DS: Data curation, Formal analysis, Investigation, Methodology, Supervision, Validation, Visualization, Writing – review & editing. SE: Data curation, Investigation, Methodology, Resources, Supervision, Writing – original draft. GA: Data curation, Formal analysis, Investigation, Project administration, Software, Supervision, Validation, Writing – original draft. KG: Conceptualization, Data curation, Formal analysis, Investigation, Methodology, Project administration, Supervision, Validation, Visualization, Writing – original draft. MA: Data curation, Formal analysis, Investigation, Methodology, Project administration, Software, Supervision, Writing – original draft. AM: Project administration, Supervision, Writing – original draft. Md: Investigation, Writing – original draft. JA: Investigation, Project administration, Writing – original draft. ID: Investigation, Writing – original draft. AP: Conceptualization, Funding acquisition, Project administration, Writing – original draft. LG: Funding acquisition, Project administration, Writing – original draft. KB: Funding acquisition, Project administration, Writing – original draft. GC: Formal analysis, Writing – original draft. AY: Investigation, Supervision, Writing – original draft. VB: Conceptualization, Funding acquisition, Methodology, Project administration, Supervision, Writing – original draft. PT: Conceptualization, Data curation, Formal analysis, Funding acquisition, Investigation, Methodology, Project administration, Resources, Software, Supervision, Validation, Visualization, Writing – original draft, Writing – review & editing. NF: Conceptualization, Data curation, Formal analysis, Funding acquisition, Investigation, Methodology, Project administration, Resources, Software, Supervision, Validation, Visualization, Writing – original draft, Writing – review & editing.

## Supplementary material

Supplementary Table 1 Panels, cells markers and fluorochrome labeling. List of flurochrome-conjugated antibodies used to analyze Treg phenotype. Abbreviation: BV510, Brilliant Violet 510; APC-R700, allophycocyanin tandem 700; BV421, Brilliant Violet 421; PE-Cy7, phycoerythrin- cyanin 7 ; PerCP-Cy5.5, peridinin-chlorophyll-protein-cyanin5.5; BV605, Brilliant Violet 605; PE, phycoerythrin; APC, allophycocyanin; AF-488, Alexa Fluor® 488; FVS, Fixable Viability Stain. BD Biosciences, USA Company for all antibodies.

Supplementary Table 2 Logistic regression analysis of the risk factors for neonatal sepsis.

Supplementary Figure 1 Gating strategies of Treg subpopulations. Cord blood mononuclear cells underwent surface staining with fluorochrome-labeled monoclonal antibodies, followed by intranuclear staining, fixation, and flow cytometric analysis. **(A)** CD4^+^ T cells were selected based on CD3 and CD4 co-expression (box plot). **(C)** Treg cells were selected based on CD25, CD127 and FOXP3 co-expression on CD3^+^CD4^+^ (box plot). **(C)** Treg cells (CD3^+^CD4^+^ CD25^+^CD127^-^FOXP3^+^) subpopulations were selected by the expression of CD39, CTLA-4, PD-1, α4β1 and α4β7, representing **(D)** Treg CD39^+^(box plot); **(E)** Treg CTLA-4^+^(box plot); **(F)** Treg PD-1^+^ (box plot); **(G)** Treg PD-1^+^ (box plot); Treg Integrin α4β1^+^(box plot) and Treg Integrin α4β1^+^ (box plot).

Supplementary Figure 2 Flow cytometry analysis of Treg cells subpopulations. Cord blood mononuclear cells underwent surface staining with fluorochrome-labeled monoclonal antibodies, followed by intranuclear staining for FOXP3, fixation, and flow cytometric analysis. (I)- **(A)** CD4^+^ T cells were selected based on CD3 and CD4 co-expression (box plot). **(B)** Treg cells were selected based on CD127 and FOXP3 co-expression on CD3^+^CD4^+^ (box plot). **(C)** Treg cell (CD3^+^CD4^+^CD127^-^FOXP3^+^) subpopulations were selected by the expression of CD45RA and FOXP3, representing CD45RA^low^FoxP3^hi^ activated Treg cells and CD45RA^hi^FoxP3^hi^ resting Treg cells (box plot). (II)- **(A)** CD4^+^ T cells were selected based on CD3 and CD4 co-expression (box plot). **(B)** Treg cells were selected based on CD25 and FOXP3 co-expression on CD3^+^CD4^+^ (box plot). **(D)** Treg cells (CD3^+^CD4^+^CD25^+^FOXP3^+^) subpopulations were selected by the expression of CD45RA and CD31, representing CD45RA^+^CD31^+^ recent thymic emigrants and CD45RA^+^CD31^-^ peripherally induced naive Treg cells (box plot).

Supplementary Figure 3 Cord blood biomarkers level in neonates subgroups of study cohort. **(A-C)** IL6; IL10 and IP-10 concentrations were measured by multiplexed assay with the Ella platform in cord blood sample obtained from the study cohort. **(D)** PCT concentrations were measured by enzyme linked fluorescent assay of mini vidas automate. **(E, F)** Box-plot of transcriptional and protein biomarkers in clinical sepsis diagnosis. CX3CR1 and CD74 mRNA level were evaluated by RT-qPCR with ABI7500 fast. Results are presented as box-plots as well as individual values in groups. EOS neonates (clinic and presumed) are compared to Hospital no sepsis neonates, as controls. The plots are shown with median and minimum/maximum values. p-values were calculated by the Mann–Whitney U-test. *p*<0.05 indicates a statistically significant difference. *p* values: **p*<0.05, ***p*<0.01, ****p*<0.001, *****p*<0.0001. ns, no statistically significant difference.

Supplementary Figure 4 Frequency and expression of homing molecules (Integrin α4β1 & Integrin α4β7) on Teff. **(A, C)** Frequency of Teff Integrin α4β1^+^ and Teff Integrin α4β7^+^cells are shown. **(B, D)** Integrin α4β1 and Integrin α4β7 expression on Teff. geometric Mean Fluorescence Intensity (gMFI) of Integrin α4β1 and Integrin α4β7 in CD4^+^CD25^+^CD127^+^ effector T cells is shown. EOS clinic (n=32), EOS presumed (n=13), Hospital no sepsis (n=41), sub-urban no sepsis (n=12). EOS neonates (clinic and presumed) are compared to Hospital no sepsis neonates, as controls. The plots are shown with median and minimum/maximum values. p-values were calculated by the Mann–Whitney U-test. *P*<0.05 indicates a statistically significant difference. *p* values: **p*<0.05, ***p*<0.01, ****p*<0.001, *****p*<0.0001. ns, no statistically significant difference.
