# Supplementary material for: Regulatory T cell homing and activation is a signature of neonatal sepsis
Source: Front Immunol. 2024 Jul 12;15:1420554. doi: 10.3389/fimmu.2024.1420554 (PMC11272980; doi:10.3389/fimmu.2024.1420554)
Supplement: Supplementary Table 1 — Panels, cells markers and fluorochrome labeling. List of flurochrome-conjugated antibodies used to analyze Treg phenotype. Abbreviation: BV510, Brilliant Violet 510; APC-R700, allophycocyanin tandem 700; BV421, Brilliant Violet 421; PE-Cy7, phycoerythrin- cyanin 7 ; PerCP-Cy5.5, peridinin-chlorophyll-protein-cyanin5.5; BV605, Brilliant Violet 605; PE, phycoerythrin; APC, allophycocyanin; AF-488, Alexa Fluor® 488; FVS, Fixable Viability Stain. BD Biosciences, USA Company for all antibodies. [file Table_1.docx]

**Supplemental Table1. Panels, cells markers and fluorochrome labeling**

| **Panel 1** | | | | **Panel 2** | | | |
| --- | --- | --- | --- | --- | --- | --- | --- |
| **Target** | **Fluorochromes** | **Clones** | **Isotypes** | **Target** | **Fluorochromes** | **Clones** | **Isotypes** |
| CD3 | BV510 | HIT3a | Ms IgG2a, κ | CD3 | BV510 | HIT3a | Ms IgG2a, κ |
| CD4 | APC-R700 | RPA-T4 | Ms IgG1, κ | CD4 | APC-R700 | RPA-T4 | Ms IgG1, κ |
| CD25 | BV421 | M-A251 | Ms BALB/c IgG1, κ | CD25 | BV421 | M-A251 | Ms BALB/c IgG1, κ |
| CD127 | PE-Cy7 | HIL-7R-M21 | Ms IgG1, κ | CD127 | PE-Cy7 | HIL-7R-M21 | Ms IgG1, κ |
| CD39 | PerCP-Cy5.5 | TU66 | Ms IgG2b, κ | CD49d | PE | 9F10 | Mouse IgG1, κ |
| CD31 | BV605 | WM59 | Ms IgG1, κ | FVS | Red | - | - |
| CD45RA | PE | HI100 | Ms IgG2b, κ | Integrinß7 | BV605 | FIB504 | CDF IgG2a, κ |
| CTLA4 (CD152) | APC | BNI3 | Ms BALB/c IgG2a, κ | PD1 (CD279) | APC |  |  |
| FOXP3 | Alexa 488 | 259D/C7 | Ms BALB/c IgG1 | FOXP3 | Alexa 488 | 259D/C7 | Ms BALB/c IgG1 |
| FVS | Red | - | - |  |  |  |  |

List of flurochrome-conjugated antibodies used to analyze Treg phenotype. Abbreviation: BV510, Brilliant Violet 510; APC-R700, allophycocyanin tandem 700; BV421, Brilliant Violet 421; PE-Cy7, phycoerythrin- cyanin 7 ; PerCP-Cy5.5, peridinin-chlorophyll-protein-cyanin5.5; BV605, Brilliant Violet 605; PE, phycoerythrin; APC, allophycocyanin; AF-488, Alexa Fluor® 488; FVS, Fixable Viability Stain. BD Biosciences, USA Company for all antibodies.
