# Supplementary material for: Regulatory T cell homing and activation is a signature of neonatal sepsis
Source: Front Immunol. 2024 Jul 12;15:1420554. doi: 10.3389/fimmu.2024.1420554 (PMC11272980; doi:10.3389/fimmu.2024.1420554)
Supplement: Supplementary Table 2 — Logistic regression analysis of the risk factors for neonatal sepsis. [file Table_2.docx]

**Supplemental Table 2. Logistic regression analysis of the risk factors for neonatal sepsis**

| Characteristics | Univariate | | Multivariate | |
| --- | --- | --- | --- | --- |
|  | _crude_ OR [95% CI] | p value | _Adjusted_ OR [95% CI] | p value |
| ***EOS Clinic*** | | | | |
| IL6 | 1.0002 [1.0; 1.0004] | 0.011 | 1.0001 [0.99; 1.0003] | 0.094 |
| IL10 | 1.004 [0.99; 1.011] | 0.298 | 0.99 [0.98; 1.01] | 0.926 |
| PCT | 1.0002 [1.0; 1.0003] | 0.001 | 1.0001 [1.0; 1.0003] | 0.039 |
| CXCL10 | 0.99 [0.99; 1.001] | 0.569 | 0.99 [0.99; 1.0004] | 0.086 |
| CX3CR1 | 1.02 [0.87; 1.19] | 0.757 | 1.17 [0.94; 1.46] | 0.148 |
| CD74 | 0.96 [0.90; 1.02] | 0.263 | 0.98 [0.90; 1.05] | 0.606 |
| Preterm birth | 1.76 [1.11; 2.81] | 0.016 | 2.94 [1.55; 5.50] | 0.001 |
| Maternal infection | 3.69 [1.89; 7.18] | < 0.001 | 2.88 [1.13; 7.38] | 0.027 |
| Maternal fever | 3.33 [2.03; 5.46] | < 0.001 | 2.64 [1.34; 5.18] | 0.005 |
| PROM | 1.69 [1.03; 2.77] | 0.037 | - | - |
| Malaria at delivery | 0.75 [0.34; 1.66] | 0.491 | 0.08 [0; 0.78] | 0.033 |
| ***EOS presumed*** | | | | |
| IL6 | 1.0001 [0.99; 1.0003] | 0.158 | 0.84 [0.62; 1.15] | 0.297 |
| IL10 | 0.99 [0.98; 1.01] | 0.739 | 1.00 [0.95; 1.05] | 0.904 |
| PCT | 1.0002 [1.0; 1.0001] | 0.001 | 1.0002 [1.0; 1.0003] | 0.016 |
| CXCL10 | 1.00 [0.99; 1.002] | 0.636 | 0.99 [0.99; 1.0002] | 0.094 |
| CX3CR1 | 1.02 [0.87; 1.20] | 0.748 | 1.18 [0.95; 1.47] | 0.142 |
| CD74 | 0.96 [0.89; 1.04] | 0.423 | 1.00 [0.90; 1.11] | 0.935 |
| Preterm birth | 1.83 [1.03; 3.24] | 0.038 | 2.68 [1.29; 5.57] | 0.008 |
| Maternal infection | 3.31 [1.49; 7.34] | 0.003 | 2.73 [0.88; 8.45] | 0.081 |
| Maternal fever | 3.33 [0.85; 3.11] | 0.134 | 1.5 [0.17; 8.90] | 0.650 |
| PROM | 2.41 [1.34; 4.34] | 0.003 | - | - |
| Malaria at delivery | 0.90 [0.35; 2.29] | 0.835 | 1.64 [0.31; 8.66] | 0.556 |
