# Supplementary figures and images for: Regulatory T cell homing and activation is a signature of neonatal sepsis
Source: Front Immunol. 2024 Jul 12;15:1420554. doi: 10.3389/fimmu.2024.1420554 (PMC11272980; doi:10.3389/fimmu.2024.1420554)

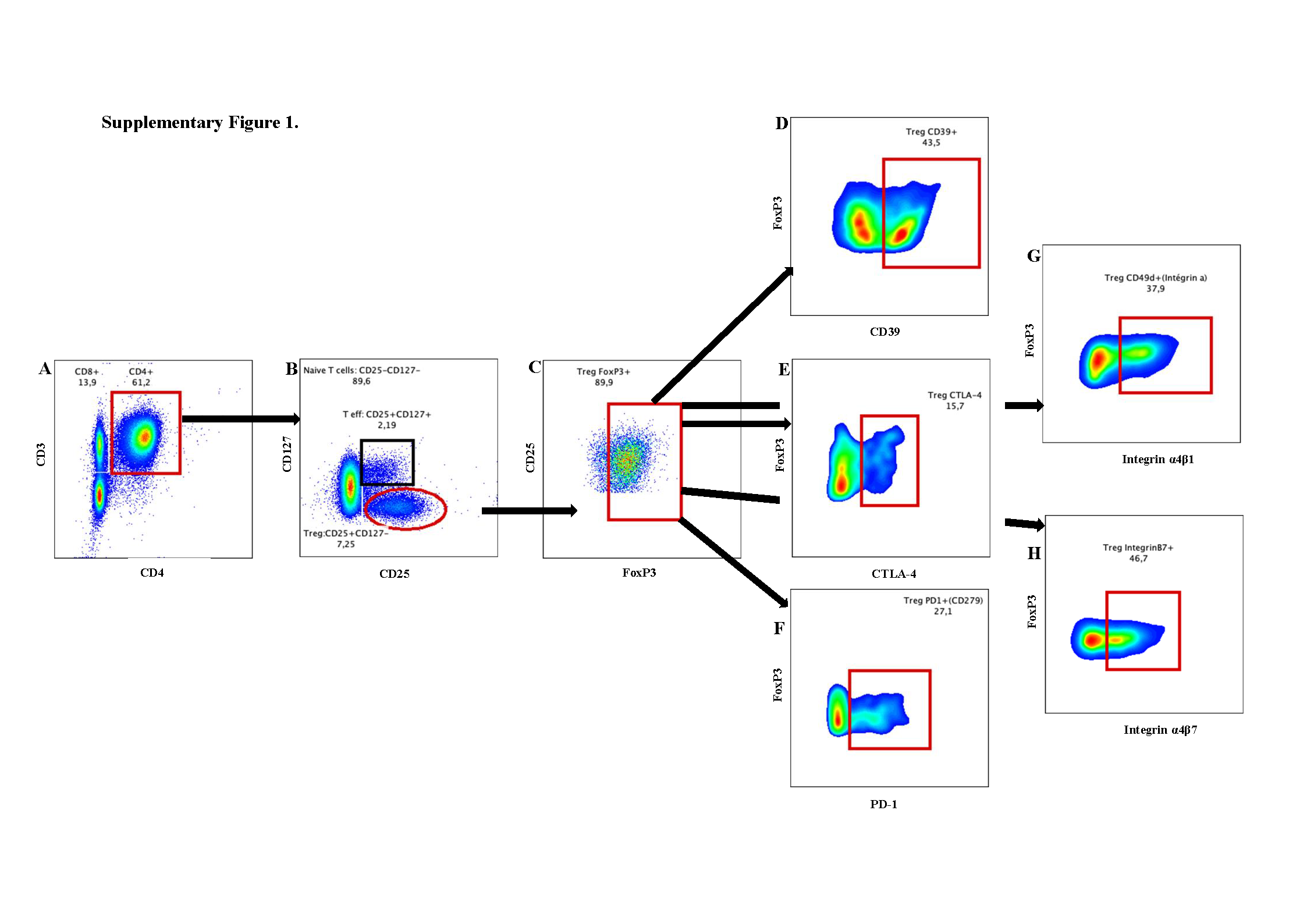

Supplement: Supplementary Figure 1 — Gating strategies of Treg subpopulations. Cord blood mononuclear cells underwent surface staining with fluorochrome-labeled monoclonal antibodies, followed by intranuclear staining, fixation, and flow cytometric analysis. (A) CD4+ T cells were selected based on CD3 and CD4 co-expression (box plot). (C) Treg cells were selected based on CD25, CD127 and FOXP3 co-expression on CD3+CD4+ (box plot). (C) Treg cells (CD3+CD4+ CD25+CD127-FOXP3+) subpopulations were selected by the expression of CD39, CTLA-4, PD-1, α4β1 and α4β7, representing (D) Treg CD39+(box plot); (E) Treg CTLA-4+(box plot); (F) Treg PD-1+ (box plot); (G) Treg PD-1+ (box plot); Treg Integrin α4β1+(box plot) and Treg Integrin α4β1+ (box plot). [file Image_1.tiff]

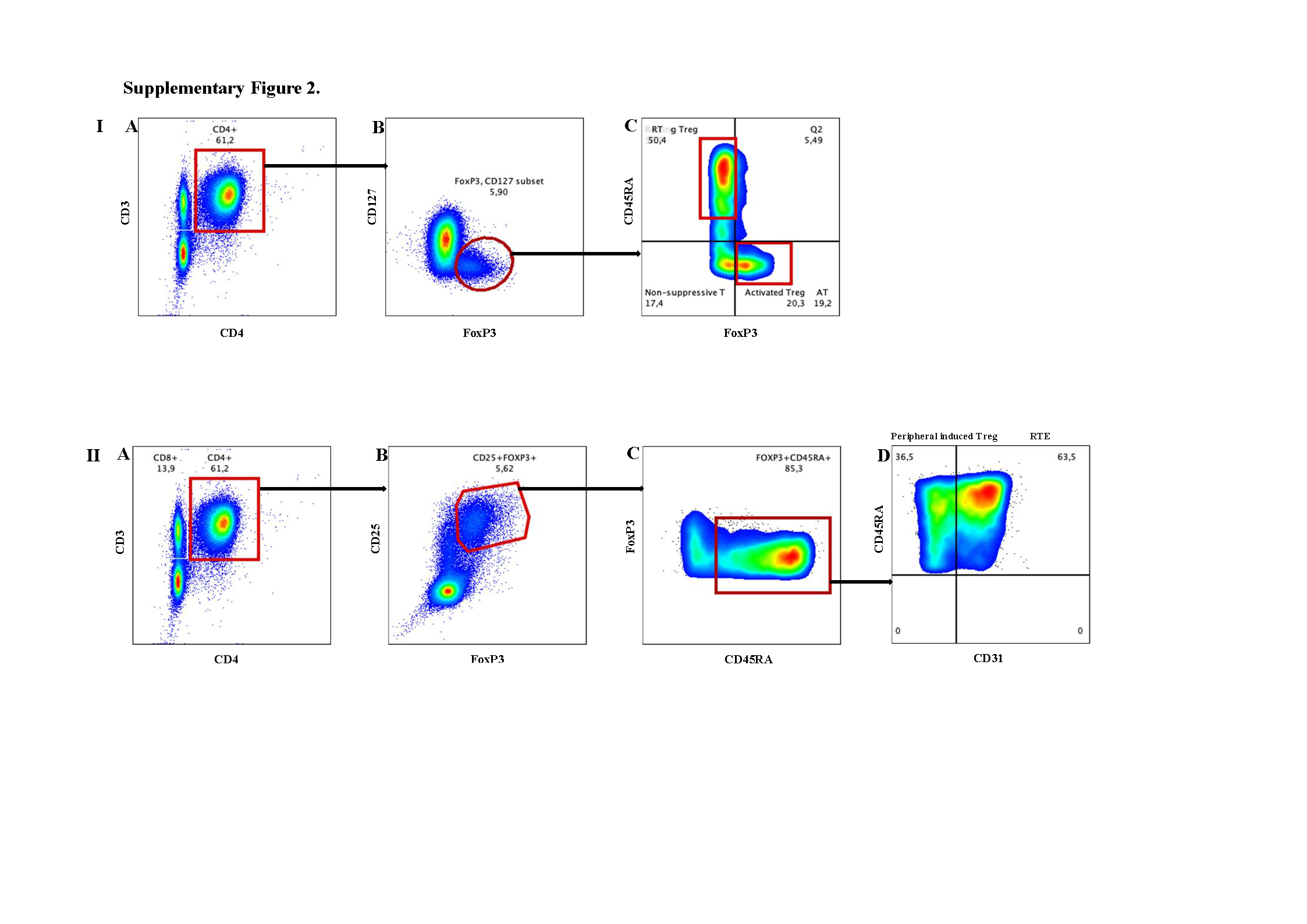

Supplement: Supplementary Figure 2 — Flow cytometry analysis of Treg cells subpopulations. Cord blood mononuclear cells underwent surface staining with fluorochrome-labeled monoclonal antibodies, followed by intranuclear staining for FOXP3, fixation, and flow cytometric analysis. (I)- (A) CD4+ T cells were selected based on CD3 and CD4 co-expression (box plot). (B) Treg cells were selected based on CD127 and FOXP3 co-expression on CD3+CD4+ (box plot). (C) Treg cell (CD3+CD4+CD127-FOXP3+) subpopulations were selected by the expression of CD45RA and FOXP3, representing CD45RAlowFoxP3hi activated Treg cells and CD45RAhiFoxP3hi resting Treg cells (box plot). (II)- (A) CD4+ T cells were selected based on CD3 and CD4 co-expression (box plot). (B) Treg cells were selected based on CD25 and FOXP3 co-expression on CD3+CD4+ (box plot). (D) Treg cells (CD3+CD4+CD25+FOXP3+) subpopulations were selected by the expression of CD45RA and CD31, representing CD45RA+CD31+ recent thymic emigrants and CD45RA+CD31- peripherally induced naive Treg cells (box plot). [file Image_2.tiff]

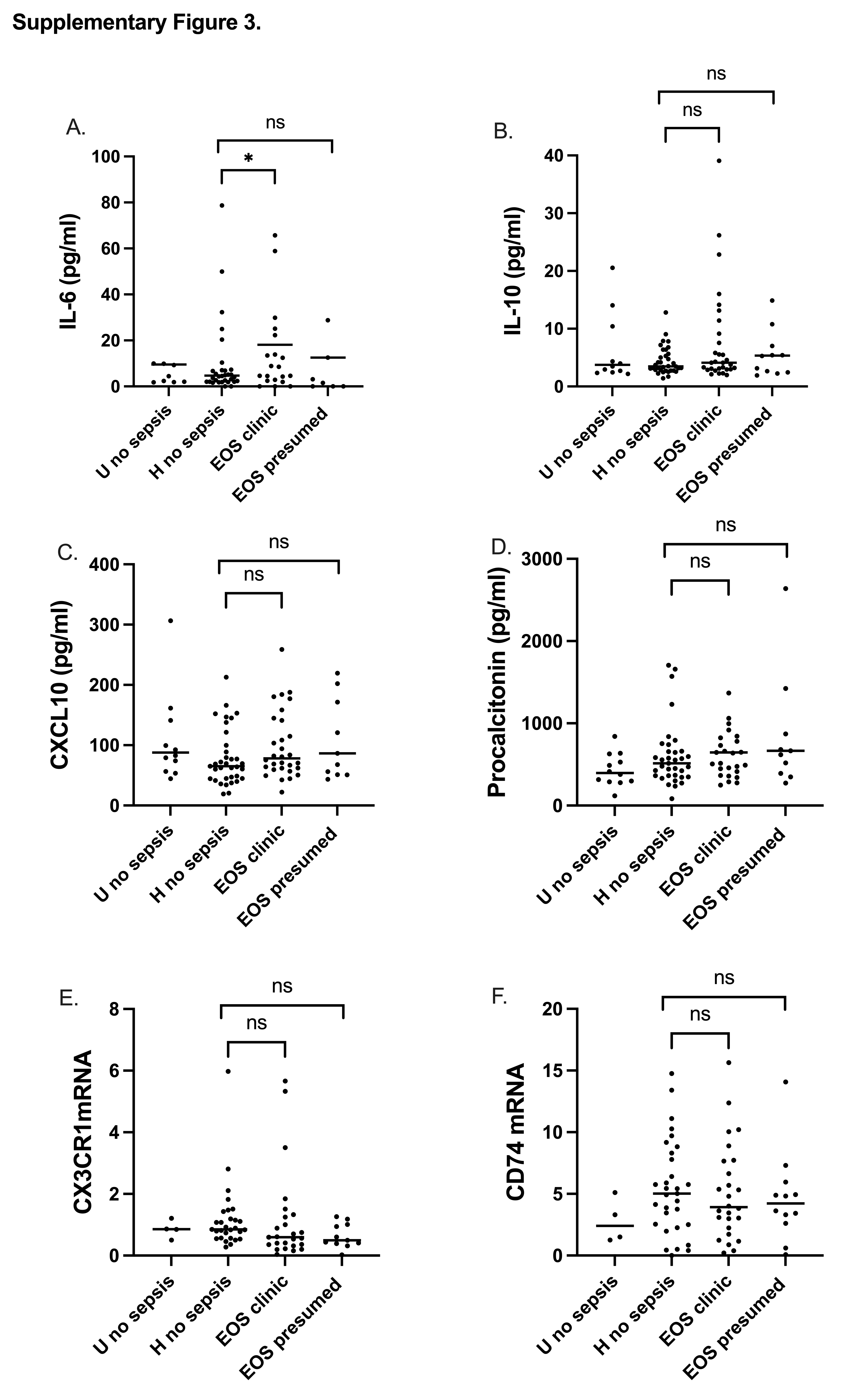

Supplement: Supplementary Figure 3 — Cord blood biomarkers level in neonates subgroups of study cohort. (A-C) IL6; IL10 and IP-10 concentrations were measured by multiplexed assay with the Ella platform in cord blood sample obtained from the study cohort. (D) PCT concentrations were measured by enzyme linked fluorescent assay of mini vidas automate. (E, F) Box-plot of transcriptional and protein biomarkers in clinical sepsis diagnosis. CX3CR1 and CD74 mRNA level were evaluated by RT-qPCR with ABI7500 fast. Results are presented as box-plots as well as individual values in groups. EOS neonates (clinic and presumed) are compared to Hospital no sepsis neonates, as controls. The plots are shown with median and minimum/maximum values. p-values were calculated by the Mann–Whitney U-test. p<0.05 indicates a statistically significant difference. p values: *p<0.05, **p<0.01, ***p<0.001, ****p<0.0001. ns, no statistically significant difference. [file Image_3.tiff]

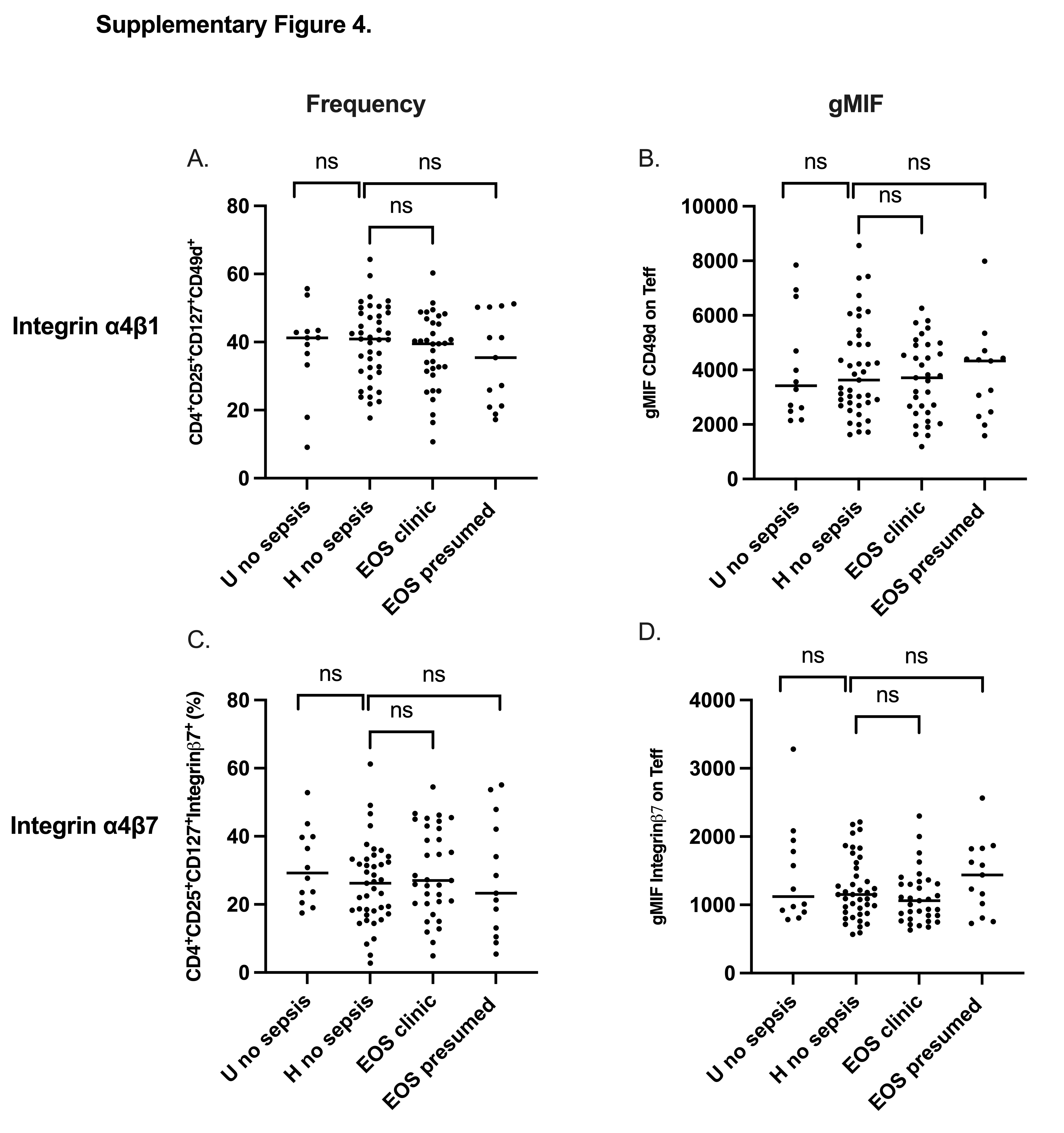

Supplement: Supplementary Figure 4 — Frequency and expression of homing molecules (Integrin α4β1 & Integrin α4β7) on Teff. (A, C) Frequency of Teff Integrin α4β1+ and Teff Integrin α4β7+cells are shown. (B, D) Integrin α4β1 and Integrin α4β7 expression on Teff. geometric Mean Fluorescence Intensity (gMFI) of Integrin α4β1 and Integrin α4β7 in CD4+CD25+CD127+ effector T cells is shown. EOS clinic (n=32), EOS presumed (n=13), Hospital no sepsis (n=41), sub-urban no sepsis (n=12). EOS neonates (clinic and presumed) are compared to Hospital no sepsis neonates, as controls. The plots are shown with median and minimum/maximum values. p-values were calculated by the Mann–Whitney U-test. P<0.05 indicates a statistically significant difference. p values: *p<0.05, **p<0.01, ***p<0.001, ****p<0.0001. ns, no statistically significant difference. [file Image_4.tiff]
